# DSCMF: prediction of LncRNA-disease associations based on dual sparse collaborative matrix factorization

**DOI:** 10.1186/s12859-020-03868-w

**Published:** 2021-05-12

**Authors:** Jin-Xing Liu, Ming-Ming Gao, Zhen Cui, Ying-Lian Gao, Feng Li

**Affiliations:** 1 School of Computer Science, Qufu Normal University, Rizhao, China; 2 Qufu Normal University Library, Qufu Normal University, Rizhao, China

**Keywords:** LncRNA-disease associations, Gaussian interaction profile kernel, Collaborative matrix factorization

## Abstract

**Background:**

In the development of science and technology, there are increasing evidences that there are some associations between lncRNAs and human diseases. Therefore, finding these associations between them will have a huge impact on our treatment and prevention of some diseases. However, the process of finding the associations between them is very difficult and requires a lot of time and effort. Therefore, it is particularly important to find some good methods for predicting lncRNA-disease associations (LDAs).

**Results:**

In this paper, we propose a method based on dual sparse collaborative matrix factorization (DSCMF) to predict LDAs. The DSCMF method is improved on the traditional collaborative matrix factorization method. To increase the sparsity, the L_2,1_-norm is added in our method. At the same time, Gaussian interaction profile kernel is added to our method, which increase the network similarity between lncRNA and disease. Finally, the AUC value obtained by the experiment is used to evaluate the quality of our method, and the AUC value is obtained by the ten-fold cross-validation method.

**Conclusions:**

The AUC value obtained by the DSCMF method is 0.8523. At the end of the paper, simulation experiment is carried out, and the experimental results of prostate cancer, breast cancer, ovarian cancer and colorectal cancer are analyzed in detail. The DSCMF method is expected to bring some help to lncRNA-disease associations research. The code can access the https://github.com/Ming-0113/DSCMF website.

## Background

In recent years, science and technology have developed rapidly, and many experts and scholars are paying more and more attention to long non-coding RNAs (lncRNAs). The length of lncRNAs is more than 200 nucleotides, and it is not involved in encoding protein functions [1]. Many experiments have demonstrated that lncRNAs play an important role in many aspects, such as epigenetic regulation, cell cycle control and cell differentiation regulation [2–4]. However, the current understanding of lncRNAs is still far from enough, and many unknown areas still need us to explore them. Therefore, we still need to strengthen the research on lncRNAs, which will also contribute to the better development of human biology.

There are increasing evidences that lncRNAs are closely linked to many human diseases, such as common cardiovascular diseases [5, 6], diabetes [7], Alzheimer's [8] and some cancers. LncRNA like MALAT1 is a transcript that is overexpressed in many cancers [9]. It is closely related to diseases such as lung cancer [10], renal cancer [11] and esophageal cancer [12]. Another example is GAS5, which is related to head and neck cancer [13], colon cancer [14], thyroid cancer [15], etc. Although some LDAs databases have been established for research by experts and scholars, the number of known LDAs in the database are far from enough, and there are many unknown associations that require people to mine them. Therefore, it is very necessary to find a method for efficient and accurate LDAs prediction.

At present, many methods have been proposed in the aspect of LDAs prediction [16]. These methods have helped more or less for predictions. For example, Sun et al. proposed a new computational model that used random walk with restart methods on the lncRNA functional similarity network [17]. A lncRNA-lncRNA functional similarity network was constructed, and the relationship between similar phenotypic diseases and functionally similar lncRNAs was used to predict novel associations. Finally, it was found through experiments that this method is indeed feasible. Chen et al. improved on the basis of the random walk with restart model, combining the disease semantic similarity matrix with the lncRNA expression similarity matrix, and setting the initial probability vector of the random walk with restart model [18]. Therefore, this model can be applied to studies of diseases without known related lncRNAs. Chen et al. proposed a Laplacian regularized least squares method to predict novel associations based on the assumption that similar diseases may be related to functionally similar lncRNAs [19]. This method was developed under the framework of semi-supervised learning and can be used to sort the candidate disease-lncRNA pairs for all diseases. Chen proposed a KATZ measurement model to predict novel LDAs by combining lncRNA expression similarity and functional similarity, as well as disease semantic similarity and GIP kernel similarity [20]. This method can predict lncRNAs with no known associations for those diseases or those with no known associations for lncRNAs. Ding et al. proposed a way to combine the gene-disease association network with the lncRNA-disease association network into a lncRNA-disease-gene tripartite graph for prediction [21]. The advantage of this method is that it can better describe the heterogeneity of coding-non-coding genes-disease associations than other methods. Ping et al. proposed a method of constructing a bipartite network to predict novel LDAs [22]. This method is based on the known topology of the lncRNA-disease network to identify those potential LDAs. Finally, the Leave-one-out cross-validation method was used to evaluate the performance of the method. Zhao et al. proposed a method for predicting novel LDAs without relying on any known lncRNA-disease association [23]. This method is based on distance correlation set that combines known lncRNA-miRNA associations and miRNA-disease associations to predict novel associations. The result proves that this method is effective and has great advantages. Ou-Yang et al. proposed a new method for predicting LDAs, called the two-side sparse self-representation method [24]. The advantage of this approach is that it can adaptively learn the self-characterization of lncRNAs and the self-characterization of diseases, a process based on the known LDAs. And this method can also be supported from the internal associations between diseases and lncRNAs. Fu et al. proposed a matrix factorization model, which mainly decomposes the data matrix of heterogeneous data sources into low-rank matrix by matrix [25].

In this paper, an improved matrix factorization model is proposed to predict LDAs. This method mainly uses the collaborative matrix factorization, and then joins the Gaussian interaction profile kernel. At the same time, the L_2,1_-norm is added to prevent over-fitting [26–28]. Since there may be some missing associations in the course of the experiment, the accuracy of our predictions will be reduced, so we also add the weight K nearest known neighbors (WKNKN) pre-processing process. The cross-validation method is used to obtain the AUC value of this method. At the end of the paper, the simulation experiment is carried out. The results show that our method is indeed superior to other methods. The specific improvements to our approach are as follows:

- In the DSCMF method, the L_2,1_-norm is introduced to sparse ${\mathbf{A}}$ and ${\mathbf{B}}$, which reduces redundant data, improves the computational power of the model, improves the robustness of the algorithm, and reduces the influence of noise on the ${\mathbf{A}}$ and ${\mathbf{B}}$ matrices.
- Network similarity is added to the DSCMF method, and we add the lncRNA network similarity matrix and the disease network similarity matrix to our method.

In the second part of this paper, we show the experimental results of the DSCMF method. The third and fourth parts discuss and summarize the DSCMF method respectively, and put forward the next work plan. The specific algorithm and detailed formula of the DSCMF method can be seen in the fifth part of this article.

## Results

### Human LncRNA-disease associations

The LncRNADisease database is a common database for studying lncRNA-disease associations [29]. This database contains 247 diseases, 369 lncRNAs and their associations. These associations were previously verified by 687 experiments [21]. The data used in this paper are 178 diseases without disease ontology (https://disease-ontology.org/) and 115 lncRNAs without expression profiles selected from ArrayExpress (https://www.ebi.ac.uk/arrayexpress/) [30]. Finally, we get a dataset with 540 lncRNA-disease associations, as listed in Table 1. ${\mathbf{Y}}$ is an adjacency matrix. If the value of this element is 1, this lncRNA $l\left( i \right)$ is related to the disease $d(j)$. Otherwise, it implies that the lncRNA has nothing to do with this disease. The ten-fold cross-validation method is applied in this paper, and the above dataset is used as the gold standard dataset for experiments to predict novel LDAs.

**Table 1:** LncRNAs, diseases, and associations in Gold Standard Dataset

Datasets	LncRNAs	Diseases	Associations
Gold standard dataset	115	178	540

### Cross validation

Cross-validation is used as an evaluation method in our experiments. And compared with the previously proposed LRLSLDA [19], ncPred [31], TPGLDA [21] and NTSHMDA [32] methods. The experiment process mainly uses the ten-fold cross-validation method. At the same time, in order to ensure the stability and reliability of our experimental results, each method is repeated 30 times. It should be noted that some unknown associations may be lost. To avoid this, the WKNKN pre-processing process is applied to our method.

At the end of the final experiment, a corresponding AUC value [33] will be generated. This AUC value is an evaluation indicator used to evaluate the quality of our method. To know the AUC value, you need to know the area under the receiver operating characteristic (ROC) curve. The AUC value is equivalent to the area under the ROC curve. ROC curve is related to true positive rate (TPR) and false positive rate (FPR). The calculation formula is as follows:

1 $$TPR = \frac{TP}{{TP + FN}},$$

2 $$FPR = \frac{FP}{{TN + FP}},$$

where $TP$ and $TN$ represent the number of positive and negative samples that are true. $FP$ and $FN$ represent the number of positive and negative samples that are false.

The area under the ROC curve is a number not greater than 1, that is, the AUC value is a number between 0 and 1. Generally, according to past experience, the AUC value is a number between 0.5 and 1. If it is less than 0.5, it proves that this method is not feasible.

### Comparison with other methods

**Fig. 1:**
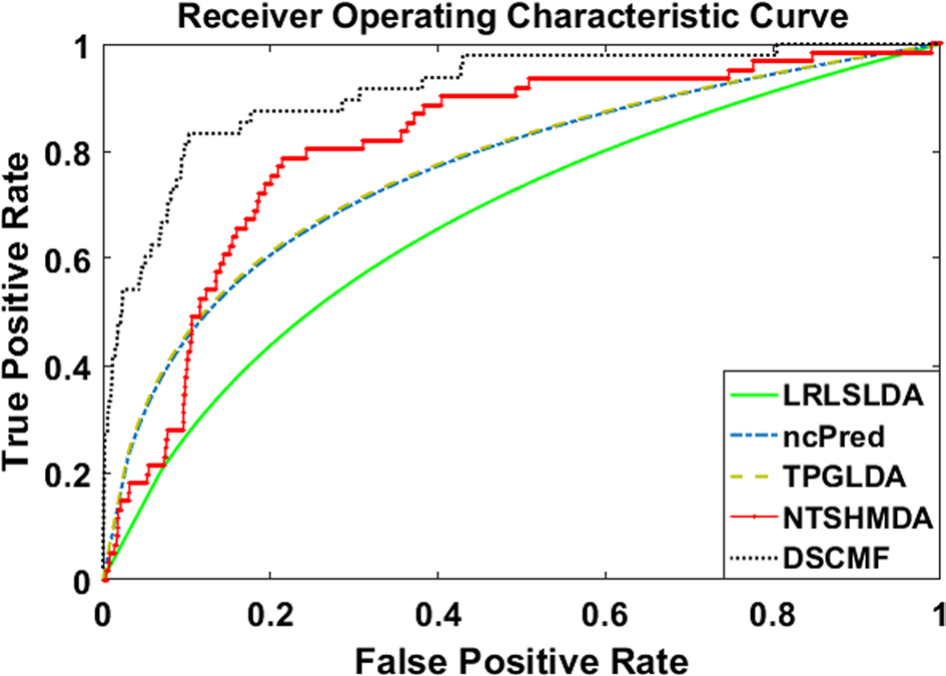
The LRLSLDA, ncPred, TPGLDA, NTSHMDA and DSCMF methods compare the performance of the AUC and ROC curves based on the ten-fold cross-validation method. It can be seen that the DSCMF method has the best performance

The experimental results of the LRLSLDA, ncPred, TPGLDA, NTSHMDA and DSCMF methods are listed in Table 2. In Table 2, we show the method with the highest AUC value and its AUC value in italics. It can be clearly seen from the experimental results that the DSCMF method has the highest AUC value, followed by the NTSHMDA method, but our method is still 5.85% higher than it. The lowest AUC value is the LRLSLDA method, which is 18.98% lower than our method. A more intuitive description of the AUC values for the various methods can be found in Fig. 1.

**Table 2:** AUC results of cross validation experiments

Methods	Gold standard dataset
LRLSLDA	0.6625 (0.0089)
ncPred	0.7566 (0.0218)
TPGLDA	0.7586 (0.0306)
NTSHMDA	0.7938 (0.0030)
*DSCMF*	*0.8523* (*0.0049*)

The above results fully show that the DSCMF method is better than the previous methods, which is more conducive to the prediction of LDAs. The DSCMF method adds a GIP kernel to the original CMF method, thereby increasing the lncRNA network similarity matrix and the disease network similarity matrix in the original method. The second is to add the L_2,1_-norm, which increases the sparsity. Therefore, this method has great advantages over other methods.

### Sensitivity analysis from WKNKN

**Fig. 2:**
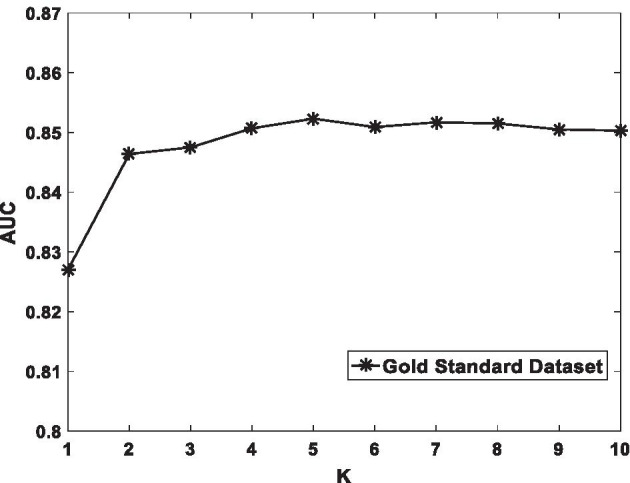
The sensitivity analysis for $K$ under CV-p

**Fig. 3:**
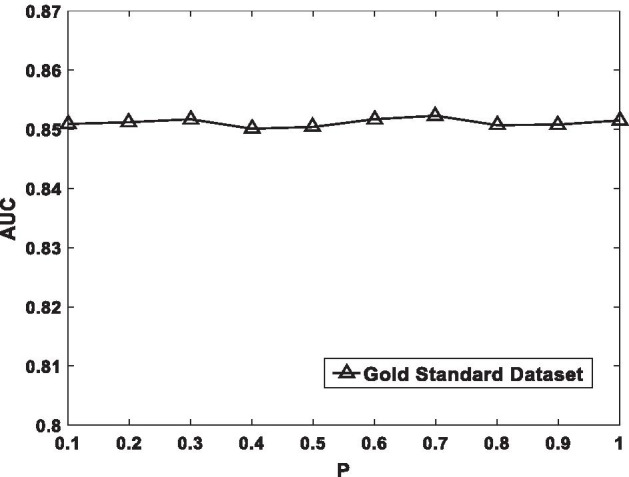
The sensitivity analysis for $P$ under CV-p

In the course of the experiment, some unknown associations that often have important influence on our prediction may be lost, so in order to avoid this negative impact will affect our experimental results, WKNKN pre-processing process is introduced in the DSCMF method. In this process, the setting of the parameters will also have a certain impact on the experimental results. Different parameters may cause the AUC value to change, so the choice of parameters is particularly important. It includes the choice of two parameters, one is the $K$ value representing the nearest known neighbor, and the other is the attenuation parameter $P$. According to previous experience, when setting $K$ to 5 and $P$ to 0.7, AUC tends to be stable. When $K$ is set to 5 and $P$ is set to 0.7, the AUC value tends to be stable. Figures 2 and 3 show the effect of the two parameters $K$ and $P$ on AUC, respectively.

### Robust analysis of DSCMF

**Fig. 4:**
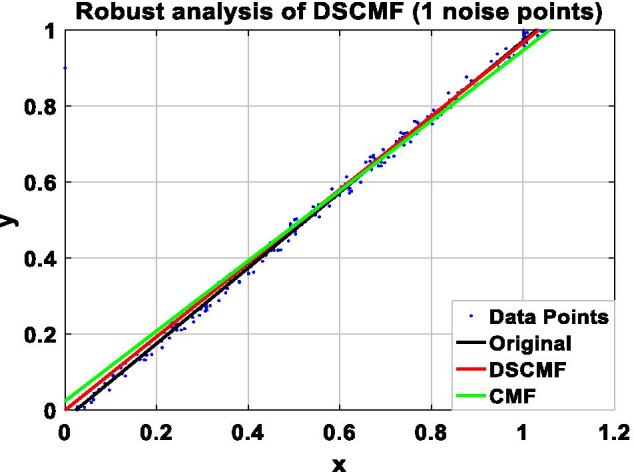
The comparison of the robustness of the CMF and DSCMF methods when the noise point is 1

**Fig. 5:**
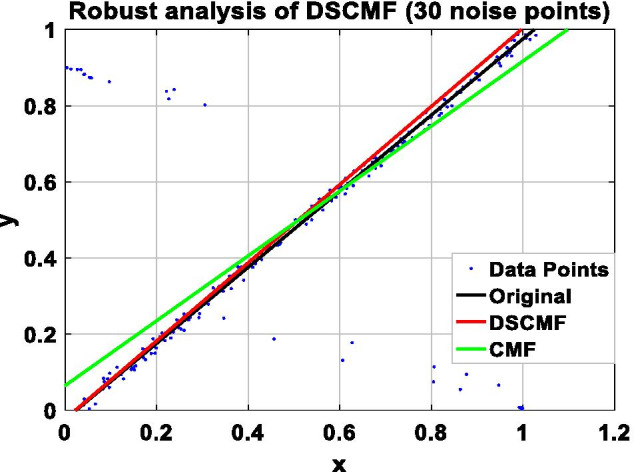
The comparison of the robustness of the CMF and DSCMF methods when the noise point is 30

**Fig. 6:**
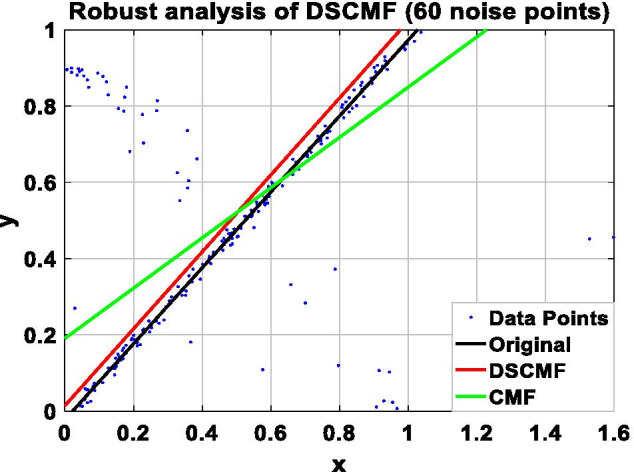
The comparison of the robustness of the CMF and DSCMF methods when the noise point is 60

**Fig. 7:**
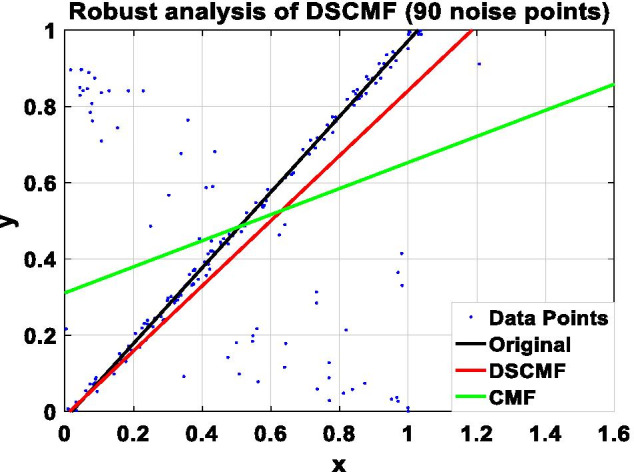
The comparison of the robustness of the CMF and DSCMF methods when the noise point is 90

In this paper, we increase the L_2,1_-norm, and the increase of the L_2,1_-norm can improve the robustness of our algorithm. In order to prove the ability of the DSCMF method to learn the subspace, that is, the anti-interference ability when restoring data is strong, the DSCMF method is applied to the synthetic dataset composed of 200 two-dimensional data points, and all the data points are distributed in a one-dimensional subspace, i.e. $y = x$. $x$ and $y$ refer to the position of the coordinate axis where the data point is located. In addition, we also apply the original CMF method to this synthetic dataset to compare with our method. The specific process is to add different numbers of noise points in the synthesized dataset to compare the robustness of the CMF and DSCMF methods. Figure 4 shows the data distribution after adding one noise point. It can be seen that both CMF and DSCMF methods can be relatively stable. Figures 5, 6, and 7 show the data distribution of 30, 60, and 90 noise points respectively. It can be clearly seen that with the increase of noise points, the DSCMF method can basically maintain a stable state, basically unaffected by noise points. However, the CMF method is more affected by noise points. It is therefore proved that the DSCMF method increases the robustness.

### Case study

In this section, simulation experiment is performed to predict some novel LDAs. For the predicted results, four common diseases are selected for research: prostate cancer, breast cancer, ovarian cancer, and colorectal cancer. The experimental procedure is as follows: For one of the diseases, the predicted score matrix obtained is sorted from high to low. Then several lncRNAs with the highest scores are selected for analysis and verified by the databases LncRNADisease and Lnc2cancer.

The first study is prostate cancer. Prostate cancer is an epithelial malignancy that is closely related to genetic factors and is present in the prostate. For more detailed information on prostate cancer, please visit the https://www.omim.org/entry/176807 website. In the original gold standard dataset, 13 lncRNAs have been shown to be associated with prostate cancer. The top 20 lncRNAs in the prediction matrix are extracted and analyzed. It is found that 12 of the original 13 lncRNAs that have been shown to be associated with prostate cancer are predicted. And in Table 3, we have indicated these 12 lncRNAs in italics. Among the remaining 8 lncRNAs, three lncRNAs, TUG1, IGF2-AS and CDKN2B-AS1, are found in the database LncRNADisease, and they are all associated with prostate cancer. Their PMIDs are 26975529 [34], 19767753 [35] and 23660942 [36], respectively. The XIST in the table is confirmed to be associated with prostate cancer in the database Lnc2cancer, and its PMID is 29212233 [37]. PTENP1, a lncRNA, is found to be associated with prostate cancer in both database LncRNADisease and Lnc2cancer. Their PMIDs are 24373479 [38] and 20577206 [39] respectively. The specific information is shown in Table 3.

**Table 3:** Predicted LncRNAs for prostate cancer

Rank	lncRNA	Evidence	Rank	lncRNA	Evidence
*1*	*MALAT1*	*Known*	*11*	*HOTTIP*	*Known*
*2*	*MEG3*	*Known*	*12*	*DANCR*	*Known*
*3*	*H19*	*Known*	13	XIST	Lnc2cancer
*4*	*HOTAIR*	*Known*	14	PTENP1	LncRNADisease; Lnc2cancer
*5*	*GAS5*	*Known*	15	TUG1	LncRNADisease
*6*	*PVT1*	*Known*	16	IGF2-AS	LncRNADisease
*7*	*UCA1*	*Known*	17	ZFAS1	Unconfirmed
*8*	*HULC*	*Known*	18	CDKN2B-AS1	LncRNADisease
*9*	*KCNQ1OT1*	*Known*	19	CCAT1	Unconfirmed
*10*	*NEAT1*	*Known*	20	SNHG16	Unconfirmed

The second disease is breast cancer. Breast cancer has become a common disease that threatens women's physical and mental health. For more detailed information about breast cancer, please visit: https://www.omim.org/entry/114480. In the gold standard dataset of the experiment, there are 20 kinds of lncRNA related to breast cancer. Comparing the predictions of the first 30 lncRNAs predicted in the simulation experiment, we find that the 17 lncRNAs in our experiment are confirmed in the gold standard dataset. These 17 lncRNAs are specifically indicated in italics in Table 4. And 2 of the remaining 13 are confirmed in the LncRNADisease database. The two lncRNAs are CCAT1 and TUG1. Their PMIDs are 26464701 [40] and 27791993 [41]. There are three lncRNAs are confirmed to be associated with breast cancer in the Lnc2cancer database, which are PTENP1, SNHG16 and TUSC7, respectively. The PMIDs of these three lncRNAs are 29085464 [42], 28232182 [43], and 23558749 [44], respectively. And KCNQ1OT1, a lncRNA, is confirmed to be associated with breast cancer in both LncRNADisease and Lnc2cancer databases. The remaining seven lncRNAs are not confirmed by the databases to be associated with breast cancer. The specific experimental results are listed in Table 4. For example, in the case of lncRNA CCAT1, previous studies have demonstrated that CCAT1 is overexpressed than normal tissue.

**Table 4:** Predicted LncRNAs for breast cancer

Rank	lncRNA	Evidence	Rank	lncRNA	Evidence
*1*	*HOTAIR*	*Known*	*16*	*ZFAS1*	*Known*
*2*	*MALAT1*	*Known*	*17*	*CDKN2B-AS1*	*Known*
*3*	*H19*	*Known*	18	CCAT1	LncRNADisease
*4*	*GAS5*	*Known*	19	PTENP1	Lnc2cancer
*5*	*UCA1*	*Known*	20	HULC	Unconfirmed
*6*	*PVT1*	*Known*	21	BANCR	Unconfirmed
*7*	*BC040587*	*Known*	22	SNHG16	Lnc2cancer
*8*	*XIST*	*Known*	23	TUG1	LncRNADisease
*9*	*MEG3*	*Known*	24	MINA	Unconfirmed
*10*	*SPRY4-IT1*	*Known*	25	TUSC7	Lnc2cancer
*11*	*CCAT2*	*Known*	26	EPB41L4A-AS1	Unconfirmed
*12*	*BCYRN1*	*Known*	27	7SK	Unconfirmed

The third disease is ovarian cancer. Ovarian cancer is a common disease in female genital organs. Its incidence is second only to cervical cancer and endometrial cancer, posing a serious threat to women's health. For more detailed information on ovarian cancer please visit https://www.omim.org/entry/167000. In the gold standard dataset, it is known that 12 lncRNAs are associated with ovarian cancer, so the top 22 lncRNAs in the prediction matrix are selected for analysis and the results are listed in Table 5. We successfully predict 11 lncRNAs, which have been confirmed in the gold standard dataset. At the same time, these 11 lncRNAs are shown in italics in Table 5. Three lncRNAs are confirmed in the LncRNADisease database, which are GAS5, NEAT1, and CCAT2, and their PMID numbers are 27779700 [45], 27608895 [46], 27558961 [47]. MEG3, SNHG16, MNX1-AS1, and ZFAS1 are confirmed to be associated with ovarian cancer in the Lnc2cancer database, and their PMIDs are 28175963 [48], 29461589 [49], 29271994 [50], and 28154416 [51], respectively. The remaining four lncRNAs are not confirmed have any association with ovarian cancer in both databases LncRNADisease and Lnc2cancer.

**Table 5:** Predicted LncRNAs for ovarian cancer

Rank	lncRNA	Evidence	Rank	lncRNA	Evidence
*1*	*HOTAIR*	*Known*	12	GAS5	LncRNADisease
*2*	*H19*	*Known*	13	MEG3	Lnc2cancer
*3*	*UCA1*	*Known*	14	NEAT1	LncRNADisease
*4*	*PVT1*	*Known*	15	SPRY4-IT1	Unconfirmed
*5*	*MALAT1*	*Known*	16	CCAT2	LncRNADisease
*6*	*XIST*	*Known*	17	HULC	Unconfirmed
*7*	*BCYRN1*	*Known*	18	SNHG16	Lnc2cancer
*8*	*CCAT1*	*Known*	19	BANCR	Unconfirmed
*9*	*SRA1*	*Known*	20	ZFAS1	Lnc2cancer
*10*	*LSINCT5*	*Known*	21	PTENP1	Unconfirmed
*11*	*MNX1-AS1*	*Known*	22	TUSC7	Unconfirmed

The last disease listed is colorectal cancer. Colorectal cancer is a common malignant tumor in humans. China is a low-incidence area for colorectal cancer, but in recent years, the incidence of colorectal cancer has increased in different regions. As can be seen from the original gold standard dataset, the dataset contains 21 lncRNAs that are associated with colorectal cancer. 20 association pairs are successfully predicted by the DSCMF algorithm, they are shown in italics in Table 6. And the remaining 10 lncRNAs are verified in the two databases LncRNADisease and Lnc2cancer whether they are associated with colorectal cancer. Among them, 4 lncRNAs are confirmed to be associated with colorectal cancer in the LncRNADisease database. These 4 lncRNAs are SPRY4-IT1, CDKN2B-AS1, TUG1 and ZFAS1, respectively. Their PMIDs are 27621655 [52], 27286457 [53], 27421138 [54] and 27461828 [55] respectively. There are also six lncRNAs that are not confirmed to be associated with colorectal cancer and further research is needed. Specific information on lncRNA and colorectal cancer is shown in Table 6:

**Table 6:** Predicted LncRNAs for colorectal cancer

Rank	lncRNA	Evidence	Rank	lncRNA	Evidence
*1*	*MALAT1*	*Known*	*16*	*TUSC7*	*Known*
*2*	*HOTAIR*	*Known*	*17*	*RPL34-AS1*	*Known*
*3*	*MEG3*	*Known*	*18*	*SNHG16*	*Known*
*4*	*GAS5*	*Known*	*19*	*MNX1-AS1*	*Known*
*5*	*PVT1*	*Known*	*20*	*NPTN-IT1*	*Known*
*6*	*UCA1*	*Known*	21	SPRY4-IT1	LncRNADisease
*7*	*H19*	*Known*	22	PTENP1	Unconfirmed
*8*	*XIST*	*Known*	23	CDKN2B-AS1	LncRNADisease
*9*	*CCAT1*	*Known*	24	LINC00261	Unconfirmed
*10*	*NEAT1*	*Known*	25	BCYRN1	Unconfirmed
*11*	*HULC*	*Known*	26	TUG1	LncRNADisease
*12*	*CCAT2*	*Known*	27	MINA	Unconfirmed
*13*	*BANCR*	*Known*	28	7SK	Unconfirmed
*14*	*LSINCT5*	*Known*	29	BC040587	Unconfirmed
*15*	*KCNQ1OT1*	*Known*	30	ZFAS1	LncRNADisease

## Discussion

Numerous studies have shown that lncRNA is indeed associated with certain diseases in humans, so it is a very important contribution to find some effective methods to predict these associations. However, the process of finding LDAs takes a long time and consumes a lot of energy. So, if you find some new ways to predict LDAs, this will be of great help to our research. The DSCMF method introduced in this paper mainly adds the L_2,1_-norm to the traditional collaborative matrix factorization method to increase the sparsity, and at the same time, the GIP kernel is used to increase the network similarity. The final cross-validation method also proves that our method is suitable for LDAs prediction. Of course, our method is not completely without disadvantages. The DSCMF method requires a long running time. Therefore, shortening the running time of our method is an important problem that we still need to solve.

## Conclusion

A ten-fold cross-validation method is used in the experimental part of this paper. And WKNKN pre-processing method is also used in the paper to solve those unknown interactions, so the accuracy of prediction is improved to the greatest extent.

In the next work, we will continue to work on this aspect of research. And, try to make up for the shortcomings in the previous research process and find some new prediction methods. At the same time, we will try to apply our method to more datasets such as miRNA-disease associations datasets, so as to more fully prove the performance of our method. At the end of the paper, I hope that the DSCMF method can be helpful for predicting lncRNA-disease associations, and we will be more committed to this research and contribute to human society.

## Methods

### LncRNA expression similarity

ArrayExpress contains more than 60,000 expression profiles of 16 human tissues, and these expression profiles are generated by RNA-Seq technology. The lncRNA expression profile used in this paper is obtained from ArrayExpress. The correlation between each pair of lncRNA expression profiles can be expressed using the Spearman correlation coefficient, which is also the similarity of lncRNA expression. The matrix ${\mathbf{S}}^{l}$ can be used to represent the lncRNA expression similarity matrix, and the expression similarity between lncRNA $l_{i}$ and lncRNA $l_{j}$ can be shown in the form of ${\mathbf{S}}^{l} \left( {l_{i} ,l_{j} } \right)$.

### Disease semantic similarity

The semantic similarity of the disease was first used in the ncRNA-disease association, and the results proved its correctness [56]. In this paper, a directed acyclic graph (DAG) is used to describe the relationship between disease semantics. For disease $D^{d}$, its directed acyclic graph can be expressed as $DAG\left( {D^{d} } \right) = \left( {D^{d} ,T\left( {D^{d} } \right),E\left( {D^{d} } \right)} \right)$, where $T\left( {D^{d} } \right)$ is represented as the set of nodes and $E\left( {D^{d} } \right)$ is represented as a set of edges between nodes. The specific formula is as follows:

3 $$DV(D^{d} ) = \sum\limits_{{d \in T(D^{d} )}}^{{}} {D_{{D^{d} }}^{{}} (d)} ,$$

4 $$D_{{D^{d} }} (d) = \left\{ {\begin{array}{*{20}c} 1 & {if\,d = D^{d} ,} \\ {\max \left\{ {\Delta * D_{{D^{d} }} \left( {d^{^{\prime}} } \right)\left| d \right.^{^{\prime}} \in children\,of\,{\text{d}}} \right\}} & {if\,{\text{d}} \ne {\text{D}}^{d} ,} \\ \end{array} } \right.$$

where $\Delta$ represents a semantic contribution factor. Given a disease semantic similarity matrix ${\mathbf{S}}^{d}$. To determine the semantic similarity between the two diseases $d_{i}$ and $d_{j}$, it is necessary to look at their common DAG parts. Therefore, as long as their DAG common parts are larger, their semantic similarities are greater. The specific calculation formula is as follows:

5 $${\mathbf{S}}^{d} (d_{i} ,d_{j} ) = \frac{{\sum\nolimits_{{t \in T(d_{i} ) \cap T(d_{j} )}} {(D_{{d_{i} }} (t) + D_{{d_{j} }} (t))} }}{{DV(d_{i} ) + DV(d_{j} )}}.$$

### Weight K nearest known neighbors

In order to prevent the loss of some unknown correlations and make our predictions more accurate, the WKNKN preprocessing process is added to the DSCMF method. In the lncRNA-disease association matrix ${\mathbf{Y}}$, if lncRNA is associated with disease, the value in the matrix is 1, otherwise it is 0. The role of pre-processing is to change these 0 or 1 to values between 0 and 1, forming a new matrix to increase the accuracy of the prediction.

### Gaussian interaction profile kernel similarity

Regardless of whether the disease is associated with the lncRNA in the lncRNA-disease network, it is likely to have a similar association with the new disease. The Gaussian interaction profile kernel similarity used in this method is based on this assumption [57]. The GIP kernel similarity can be used in this method to represent the network topological structure of LDAs. The topological structure of lncRNA $l_{i}$, $l_{j}$ and disease $d_{i}$, $d_{j}$ are represented by the following formula:

6 $$GIP_{lncRNA} (l_{i,} l_{j} ) = \exp ( - \gamma \left\| {{\mathbf{Y}}(l_{i} ) - {\mathbf{Y}}(l_{j} )} \right\|^{2} ),$$

7 $$GIP_{disease} (d_{i,} d_{j} ) = \exp ( - \gamma \left\| {{\mathbf{Y}}(d_{i} ) - {\mathbf{Y}}(d_{j} )} \right\|^{2} ).$$

The parameters of the adjustment kernel bandwidth represented by $\gamma$ in the above two formulas. ${\mathbf{Y}}(l_{i} )$ stands for a binary vector, the $i$-th row of ${\mathbf{Y}}$, which represents the interaction profiles of the association between $l_{i}$ and each disease. Next, the lncRNA expression similarity matrix and the network similarity matrix are combined by using formula (8). Similarly, the disease semantic similarity matrix and the network similarity matrix are combined by using formula (9).

8 $${\mathbf{K}}_{l} {\mathbf{ = }}\alpha {\mathbf{S}}^{l} + (1 - \alpha ){\mathbf{GIP}}_{l} ,$$

9 $${\mathbf{K}}_{d} {\mathbf{ = }}\alpha {\mathbf{S}}^{d} + (1 - \alpha ){\mathbf{GIP}}_{d} .$$

In the above two formulas, $\alpha \in \left[ {0,1} \right]$, and it is a parameter that can be adjusted. Where ${\mathbf{K}}_{l}$ is the final matrix combining lncRNA expression similarity and network similarity, and ${\mathbf{K}}_{d}$ is the final matrix that combines the semantic similarity of disease with network similarity.

### DSCMF

Collaborative filtering is introduced in the traditional CMF method [58], which can accurately predict some novel LDAs. The objective function of the traditional CMF is as follows:

10 $$\begin{aligned} & \min\nolimits_{{{\mathbf{A}},{\mathbf{B}}}} = ||{\mathbf{Y}} - {\mathbf{AB}}^{T} ||_{F}^{2} + \lambda_{h} (||{\mathbf{A}}||_{F}^{2} + ||{\mathbf{B}}||_{F}^{2} ) \\ & \quad + \lambda_{l} ||{\mathbf{S}}^{l} - {\mathbf{AA}}^{\text{T}} ||_{F}^{2} + \lambda_{d} ||{\mathbf{S}}^{d} - {\mathbf{BB}}^{T} ||_{F}^{2} , \\ \end{aligned}$$

where $\left\| \cdot \right\|_{F}$ is Frobenius norm. $\lambda_{h}$, $\lambda_{l}$ and $\lambda_{d}$ are positive parameters.

Then, the ${\mathbf{S}}^{l}$ in the traditional collaborative matrix factorization method is replaced by ${\mathbf{K}}_{l}$. Similarly, ${\mathbf{S}}^{d}$ is replaced by ${\mathbf{K}}_{d}$, thereby increasing the network similarity between lncRNA and disease. The improved formula is as follows:

11 $$\begin{aligned} & \min\nolimits_{{{\mathbf{A}},{\mathbf{B}}}} = ||{\mathbf{Y}} - {\mathbf{AB}}^{T} ||_{F}^{2} + \lambda_{h} (||{\mathbf{A}}||_{F}^{2} + ||{\mathbf{B}}||_{F}^{2} ) \\ & \quad + \lambda_{l} ||{\mathbf{K}}_{l} - {\mathbf{AA}}^{\text{T}} ||_{F}^{2} + \lambda_{d} ||{\mathbf{K}}_{d} - {\mathbf{BB}}^{T} ||_{F}^{2} . \\ \end{aligned}$$

At the same time, to increase the sparsity, the method in this paper adds L_2,1_-norm to matrix ${\mathbf{A}}$ and ${\mathbf{B}}$ respectively. The final objective function can be written as:

12 $$\begin{aligned} & \min\nolimits_{{{\mathbf{A}},{\mathbf{B}}}} = ||{\mathbf{Y}} - {\mathbf{AB}}^{\text{T}} ||_{F}^{2} + \lambda_{h} (||{\mathbf{A}}||_{F}^{2} + ||{\mathbf{B}}||_{F}^{2} ) + \lambda_{h} ||{\mathbf{A}}||_{2,1} \\ & \quad + \lambda_{h} ||{\mathbf{B}}||_{2,1} + \lambda_{l} ||{\mathbf{K}}_{l} - {\mathbf{AA}}^{\text{T}} ||_{F}^{2} + \lambda_{d} ||{\mathbf{K}}_{d} - {\mathbf{BB}}^{\text{T}} ||_{F}^{2} . \\ \end{aligned}$$

The matrices ${\mathbf{A}}$ and ${\mathbf{B}}$ of this formula are two latent feature matrices produced by the decomposition of the matrix ${\mathbf{Y}}$. Where $\left\| {\mathbf{A}} \right\|_{F}^{2} = Tr\left( {{\mathbf{A}}^{T} {\mathbf{A}}} \right) = Tr\left( {{\mathbf{AA}}^{T} } \right)$, $\left\| {\mathbf{A}} \right\|_{2,1} = Tr\left( {{\mathbf{A}}^{T} {\mathbf{D}}_{1} {\mathbf{A}}} \right)$ and $\left\| {\mathbf{B}} \right\|_{2,1} = Tr\left( {{\mathbf{B}}^{T} {\mathbf{D}}_{2} {\mathbf{B}}} \right)$.${\mathbf{D}}_{1}$, ${\mathbf{D}}_{2}$ are two diagonal matrices, where the values of the $j$-th diagonal element are denoted as $d_{jj}^{1} = {1 \mathord{\left/ {\vphantom {1 {2||\left( {\mathbf{A}} \right)^{j} ||}}} \right. \kern-\nulldelimiterspace} {2||\left( {\mathbf{A}} \right)^{j} ||}}_{2}$, $d_{jj}^{2} = {1 \mathord{\left/ {\vphantom {1 {2||\left( {\mathbf{B}} \right)^{j} ||}}} \right. \kern-\nulldelimiterspace} {2||\left( {\mathbf{B}} \right)^{j} ||}}_{2}$, respectively.

**Fig. 8:**
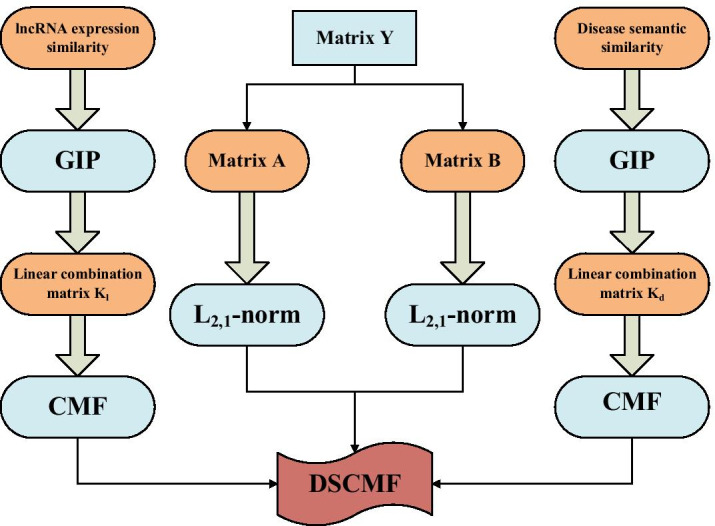
Method flow chart. The DSCMF method consists of two parts. First, the matrix ${\mathbf{Y}}$ is decomposed into ${\mathbf{A}}$ and ${\mathbf{B}}$, and L_2,1_-norm is added to ${\mathbf{A}}$ and ${\mathbf{B}}$, respectively. Second is to join the GIP kernel in the CMF method

The first term is to construct an approximate model, the purpose is to find the matrix ${\mathbf{A}}$ and ${\mathbf{B}}$. The second part is to add the Tikhonov regularization terms to prevent overfitting. The third part is to add the L_2,1_-norm to matrix ${\mathbf{A}}$. The fourth part is to add the L_2,1_-norm to matrix ${\mathbf{B}}$.The last two parts are the collaborative regularization terms of lncRNA expression similarity matrix and disease semantic similarity matrix. A detailed flow chart of the DSCMF method is shown in Fig. 8.

### Optimization and algorithm of DSCMF method

In this paper, we use the least squares method to update ${\mathbf{A}}$ and ${\mathbf{B}}$ to optimize the new method of this paper. In the first step, the values of ${\mathbf{A}}$ and ${\mathbf{B}}$ need to be initialized, so the singular value decomposition (SVD) method is used in this paper. The initial formula is:

13 $$\left[ {{\mathbf{U}},{\mathbf{S}},{\mathbf{V}}} \right] = SVD({\mathbf{Y}},k),{\mathbf{A}} = {\mathbf{US}}_{k}^{{{\raise0.7ex\hbox{$1$} \!\mathord{\left/ {\vphantom {1 2}}\right.\kern-\nulldelimiterspace} \!\lower0.7ex\hbox{$2$}}}} ,{\mathbf{B}} = {\mathbf{VS}}_{k}^{{{\raise0.7ex\hbox{$1$} \!\mathord{\left/ {\vphantom {1 2}}\right.\kern-\nulldelimiterspace} \!\lower0.7ex\hbox{$2$}}}} ,$$

where ${\mathbf{S}}_{k}$ represents a diagonal matrix that contains the $k$ largest singular values. Next, based on the objective function, the partial derivatives are obtained for ${\mathbf{A}}$ and ${\mathbf{B}}$, respectively, and their partial derivatives are zero. Finally, updating is stopped once ${\mathbf{A}}$ and ${\mathbf{B}}$ converge. The iteration formula is as follows:

14 $${\mathbf{A}} = \left( {{\mathbf{YB}} + \lambda_{l} {\mathbf{K}}_{l} {\mathbf{A}}} \right)\left( {{\mathbf{B}}^{\text{T}} {\mathbf{B}} + \lambda_{h} {\mathbf{I}}_{\text{k}} + \lambda_{l} {\mathbf{A}}^{\text{T}} {\mathbf{A}} + \lambda_{h} {\mathbf{D}}_{1} {\mathbf{I}}_{K} } \right)^{ - 1} ,$$

15 $${\mathbf{B}} = \left( {{\mathbf{Y}}^{\text{T}} {\mathbf{A}} + \lambda_{d} {\mathbf{K}}_{\text{d}} {\mathbf{B}}} \right)\left( {{\mathbf{A}}^{\text{T}} {\mathbf{A}} + \lambda_{h} {\mathbf{I}}_{\text{k}} + \lambda_{d} {\mathbf{B}}^{\text{T}} {\mathbf{B}} + \lambda_{h} {\mathbf{D}}_{2} {\mathbf{I}}_{\text{k}} } \right)^{ - 1} ,$$

where $\lambda_{h}$, $\lambda_{l}$ and $\lambda_{d}$ are a combination of the best parameters automatically selected from $\lambda_{h} \in \left\{ {2^{ - 2} ,2^{ - 1} ,2^{0} ,2^{1} } \right\}$ and ${{\lambda_{l} } \mathord{\left/ {\vphantom {{\lambda_{l} } {\lambda_{d} }}} \right. \kern-\nulldelimiterspace} {\lambda_{d} }} \in \left\{ {0,10^{ - 4} ,10^{ - 3} ,10^{ - 2} ,10^{ - 1} } \right\}$.

**Fig. 9:**
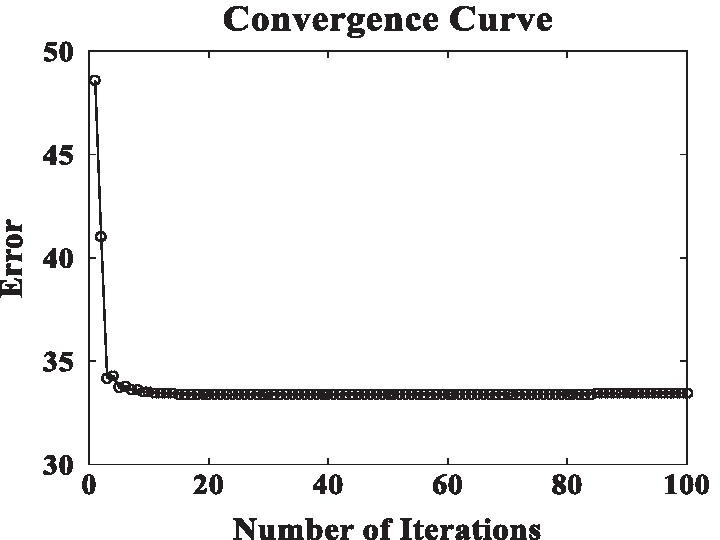
Convergence curve of the DSCMF method. When the number of iterations is about ten, our method tends to converge

Through the detailed description of the above process, the algorithm of the DSCMF method can be organized as follows:
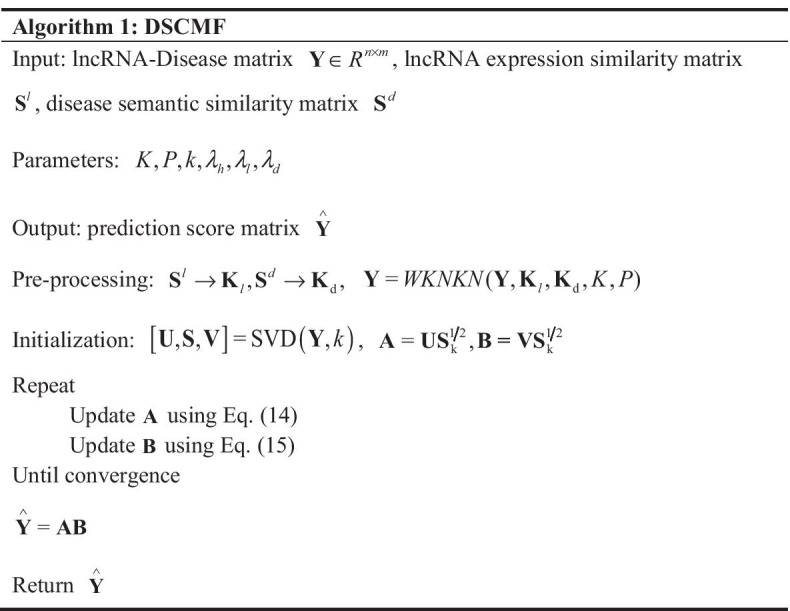


The DSCMF method is convergent. The maximum number of iterations is set to 100 times during the experiment, in order to find the local optimal solution of the objective function. The convergence curve is shown in Fig. 9. The algorithm tends to converge in about 10 times, which proves that our algorithm can converge quickly.

## Data Availability

The datasets that support the findings of this study are available in https://www.cuilab.cn/lncrnadisease.

## Abbreviations

DSCMF: Dual sparse collaborative matrix factorization
GIP: Gaussian interaction profile
WKNKN: Weighted K nearest known neighbors
CV: Cross-validation
SVD: Singular value decomposition

## References

[1] Ponting CP, Oliver PL, Reik W (2009). Evolution and functions of long noncoding RNAs. Cell.

[2] Wang C, Wang L, Ding Y, Lu X, Zhang G, Yang J, Zheng H, Wang H, Jiang Y, Xu L (2017). LncRNA structural characteristics in epigenetic regulation. Int J Mol Sci.

[3] Wapinski O, Chang HY (2011). Long noncoding RNAs and human disease. Trends Cell Biol.

[4] Alvarez-Dominguez JR, Hu W, Lodish HF. Regulation of eukaryotic cell differentiation by long non-coding RNAs. In: Molecular biology of long non-coding RNAs. Springer; 2013. p. 15–67.

[5] Micheletti R, Plaisance I, Abraham BJ, Sarre A, Ting C-C, Alexanian M, Maric D, Maison D, Nemir M, Young RA (2017). The long noncoding RNA Wisper controls cardiac fibrosis and remodeling. Sci Transl Med.

[6] Zhu YP, Hedrick CC, Gaddis DE (2017). Hematopoietic stem cells gone rogue. Science.

[7] Bai X, Geng J, Li X, Wan J, Liu J, Zhou Z, Liu X (2018). Long noncoding RNA LINC01619 regulates microRNA-27a/forkhead box protein O1 and endoplasmic reticulum stress-mediated podocyte injury in diabetic nephropathy. Antioxid Redox Signal.

[8] Luo Q, Chen Y (2016). Long noncoding RNAs and Alzheimer’s disease. Clin Interv Aging.

[9] Änkö M-L, Neugebauer KM (2010). Long noncoding RNAs add another layer to pre-mRNA splicing regulation. Mol Cell.

[10] Guo F, Yu F, Wang J, Li Y, Li Y, Li Z, Zhou Q (2015). Expression of MALAT1 in the peripheral whole blood of patients with lung cancer. Biomed Rep.

[11] Xiao H, Tang K, Liu P, Chen K, Hu J, Zeng J, Xiao W, Yu G, Yao W, Zhou H (2015). LncRNA MALAT1 functions as a competing endogenous RNA to regulate ZEB2 expression by sponging miR-200s in clear cell kidney carcinoma. Oncotarget.

[12] Huang C, Yu Z, Yang H, Lin Y (2016). Increased MALAT1 expression predicts poor prognosis in esophageal cancer patients. Biomed Pharmacother.

[13] Fayda M, Isin M, Tambas M, Guveli M, Meral R, Altun M, Sahin D, Ozkan G, Sanli Y, Isin H (2016). Do circulating long non-coding RNAs (lncRNAs)(LincRNA-p21, GAS 5, HOTAIR) predict the treatment response in patients with head and neck cancer treated with chemoradiotherapy?. Tumor Biol.

[14] Lucafò M, Di Silvestre A, Romano M, Avian A, Antonelli R, Martelossi S, Naviglio S, Tommasini A, Stocco G, Ventura A (2018). Role of the long non-coding rna growth arrest-specific 5 in glucocorticoid response in children with inflammatory bowel disease. Basic Clin Pharmacol Toxicol.

[15] Guo L-J, Zhang S, Gao B, Jiang Y, Zhang X-H, Tian W-G, Hao S, Zhao J-J, Zhang G, Hu C-Y (2017). Low expression of long non-coding RNA GAS5 is associated with poor prognosis of patients with thyroid cancer. Exp Mol Pathol.

[16] Cui Z, Liu J-X, Gao Y-L, Zhu R, Yuan S-S (2019). LncRNA-disease associations prediction using bipartite local model with nearest profile-based association inferring. IEEE J Biomed Health Inform.

[17] Sun J, Shi H, Wang Z, Zhang C, Liu L, Wang L, He W, Hao D, Liu S, Zhou M (2014). Inferring novel lncRNA-disease associations based on a random walk model of a lncRNA functional similarity network. Mol BioSyst.

[18] Chen X, You Z, Yan G, Gong D (2016). IRWRLDA: improved random walk with restart for lncRNA-disease association prediction. Oncotarget.

[19] Chen X, Yan G-Y (2013). Novel human lncRNA-disease association inference based on lncRNA expression profiles. Bioinformatics.

[20] Chen X (2015). KATZLDA: KATZ measure for the lncRNA-disease association prediction. Sci Rep.

[21] Ding L, Wang M, Sun D, Li A (2018). TPGLDA: Novel prediction of associations between lncRNAs and diseases via lncRNA-disease-gene tripartite graph. Sci Rep.

[22] Ping P, Wang L, Kuang L, Ye S, Iqbal MFB, Pei T (2018). A novel method for lncRNA-disease association prediction based on an lncRNA-disease association network. IEEE/ACM Trans Comput Biol Bioinform.

[23] Zhao H, Kuang L, Wang L, Xuan Z (2018). A novel approach for predicting disease-lncRNA associations based on the distance correlation set and information of the miRNAs. Comput Math Methods Med..

[24] Ou-Yang L, Huang J, Zhang X-F, Li Y-R, Sun Y, He S, Zhu Z (2019). LncRNA-disease association prediction using two-side sparse self-representation. Front Genet.

[25] Fu G, Wang J, Domeniconi C, Yu G (2017). Matrix factorization-based data fusion for the prediction of lncRNA–disease associations. Bioinformatics.

[26] Cui Z, Gao Y-L, Liu J-X, Dai L-Y, Yuan S-S (2019). L 2, 1-GRMF: an improved graph regularized matrix factorization method to predict drug-target interactions. BMC Bioinform.

[27] Cui Z, Gao Y-L, Liu J-X, Wang J, Shang J, Dai L-Y (2019). The computational prediction of drug-disease interactions using the dual-network L 2, 1-CMF method. BMC Bioinform.

[28] Gao M-M, Cui Z, Gao Y-L, Liu J-X, Zheng C-H (2019). Dual-network sparse graph regularized matrix factorization for predicting miRNA-disease associations. Mol Omics.

[29] Chen G, Wang Z, Wang D, Qiu C, Liu M, Chen X, Zhang Q, Yan G, Cui Q (2012). LncRNADisease: a database for long-non-coding RNA-associated diseases. Nucleic Acids Res.

[30] Parkinson H, Kapushesky M, Shojatalab M, Abeygunawardena N, Coulson R, Farne A, Holloway E, Kolesnykov N, Lilja P, Lukk M (2006). ArrayExpress—a public database of microarray experiments and gene expression profiles. Nucleic Acids Res.

[31] Alaimo S, Giugno R, Pulvirenti A (2014). ncPred: ncRNA-disease association prediction through tripartite network-based inference. Front Bioeng Biotechnol.

[32] Luo J, Long Y (2020). NTSHMDA: prediction of human microbe-disease association based on random walk by integrating network topological similarity. IEEE/ACM Trans Comput Biol Bioinform..

[33] Huang J, Ling CX (2005). Using AUC and accuracy in evaluating learning algorithms. IEEE Trans Knowl Data Eng.

[34] Du Z, Sun T, Hacisuleyman E, Fei T, Wang X, Brown M, Rinn JL, Lee MG-S, Chen Y, Kantoff PW (2016). Integrative analyses reveal a long noncoding RNA-mediated sponge regulatory network in prostate cancer. Nat Commun.

[35] Eeles RA, Kote-Jarai Z, Al Olama AA, Giles GG, Guy M, Severi G, Muir K, Hopper JL, Henderson BE, Haiman CA (2009). Identification of seven new prostate cancer susceptibility loci through a genome-wide association study. Nat Genet.

[36] Cheetham S, Gruhl F, Mattick J, Dinger M (2013). Long noncoding RNAs and the genetics of cancer. Br J Cancer.

[37] Du Y, Weng X-D, Wang L, Liu X-H, Zhu H-C, Guo J, Ning J-Z, Xiao C-C (2017). LncRNA XIST acts as a tumor suppressor in prostate cancer through sponging miR-23a to modulate RKIP expression. Oncotarget.

[38] Martens-Uzunova ES, Böttcher R, Croce CM, Jenster G, Visakorpi T, Calin GA (2014). Long noncoding RNA in prostate, bladder, and kidney cancer. Eur Urol.

[39] Poliseno L, Salmena L, Zhang J, Carver B, Haveman WJ, Pandolfi PP (2010). A coding-independent function of gene and pseudogene mRNAs regulates tumour biology. Nature.

[40] Zhang X-F, Liu T, Li Y, Li S (2015). Overexpression of long non-coding RNA CCAT1 is a novel biomarker of poor prognosis in patients with breast cancer. Int J Clin Exp Pathol.

[41] Zhao X-B, Ren G-S. WITHDRAWN: LncRNA TUG1 promotes breast cancer cell proliferation via inhibiting miR-9. Cancer Biomark Sect A Dis Markers. 2016. 10.3233/CBM-160669 27791993

[42] Chen S, Wang Y, Zhang J-H, Xia Q-J, Sun Q, Li Z-K, Zhang J-G, Tang M-S, Dong M-S (2017). Long non-coding RNA PTENP1 inhibits proliferation and migration of breast cancer cells via AKT and MAPK signaling pathways. Oncol Lett.

[43] Cai C, Huo Q, Wang X, Chen B, Yang Q (2017). SNHG16 contributes to breast cancer cell migration by competitively binding miR-98 with E2F5. Biochem Biophys Res Commun.

[44] Liu Q, Huang J, Zhou N, Zhang Z, Zhang A, Lu Z, Wu F, Mo Y-Y (2013). LncRNA loc285194 is a p53-regulated tumor suppressor. Nucleic Acids Res.

[45] Li J, Huang H, Li Y, Li L, Hou W, You Z (2016). Decreased expression of long non-coding RNA GAS5 promotes cell proliferation, migration and invasion, and indicates a poor prognosis in ovarian cancer. Oncol Rep.

[46] Chen Z, Zhang Z, Xie B, Zhang H (2016). Clinical significance of up-regulated lncRNA NEAT1 in prognosis of ovarian cancer. Eur Rev Med Pharmacol Sci.

[47] Zheng J, Zhao S, He X, Zheng Z, Bai W, Duan Y, Cheng S, Wang J, Liu X, Zhang G (2016). The up-regulation of long non-coding RNA CCAT2 indicates a poor prognosis for prostate cancer and promotes metastasis by affecting epithelial-mesenchymal transition. Biochem Biophys Res Commun.

[48] Zhang J, Liu J, Xu X, Li L (2017). Curcumin suppresses cisplatin resistance development partly via modulating extracellular vesicle-mediated transfer of MEG3 and miR-214 in ovarian cancer. Cancer Chemother Pharmacol.

[49] Yang X, Wang G, Luo L (2018). Long non-coding RNA SNHG16 promotes cell growth and metastasis in ovarian cancer. Eur Rev Med Pharmacol Sci.

[50] Li A, Zhang H (2017). Overexpression of lncRNA MNX1-AS1 is associated with poor clinical outcome in epithelial ovarian cancer. Eur Rev Med Pharmacol Sci.

[51] Liu R, Zeng Y, Zhou C-F, Wang Y, Li X, Liu Z-Q, Chen X-P, Zhang W, Zhou H-H (2017). Long noncoding RNA expression signature to predict platinum-based chemotherapeutic sensitivity of ovarian cancer patients. Sci Rep.

[52] Cao D, Ding Q, Yu W, Gao M, Wang Y (2016). Long noncoding RNA SPRY4-IT1 promotes malignant development of colorectal cancer by targeting epithelial–mesenchymal transition. OncoTargets Ther.

[53] Sun Z, Ou C, Ren W, Xie X, Li X, Li G (2016). Downregulation of long non-coding RNA ANRIL suppresses lymphangiogenesis and lymphatic metastasis in colorectal cancer. Oncotarget.

[54] Wang L, Zhao Z, Feng W, Ye Z, Dai W, Zhang C, Peng J, Wu K (2016). Long non-coding RNA TUG1 promotes colorectal cancer metastasis via EMT pathway. Oncotarget.

[55] Wang W, Xing C (2016). Upregulation of long noncoding RNA ZFAS1 predicts poor prognosis and prompts invasion and metastasis in colorectal cancer. Pathol Res Pract.

[56] Chen X, Yan CC, Luo C, Ji W, Zhang Y, Dai Q (2015). Constructing lncRNA functional similarity network based on lncRNA-disease associations and disease semantic similarity. Sci Rep.

[57] van Laarhoven T, Nabuurs SB, Marchiori E (2011). Gaussian interaction profile kernels for predicting drug–target interaction. Bioinformatics.

[58] Shen Z, Zhang Y-H, Han K, Nandi AK, Honig B, Huang D-S (2017). miRNA-disease association prediction with collaborative matrix factorization. Complexity..

